# Portfolio Optimization with a Mean-Entropy-Mutual Information Model

**DOI:** 10.3390/e24030369

**Published:** 2022-03-04

**Authors:** Rodrigo Gonçalves Novais, Peter Wanke, Jorge Antunes, Yong Tan

**Affiliations:** 1 COPPEAD Graduate Business School, Federal University of Rio de Janeiro, Rio de Janeiro 21941-918, Brazil; novaisrodrigo@outlook.com (R.G.N.); peter@coppead.ufrj.br (P.W.); jorge.moreira@coppead.ufrj.br (J.A.); 2 School of Management, University of Bradford, Bradford BD7 1DP, West Yorkshire, UK

**Keywords:** portfolio optimization, entropy, mutual information, variance and covariance

## Abstract

This paper describes a new model for portfolio optimization (PO), using entropy and mutual information instead of variance and covariance as measurements of risk. We also compare the performance in and out of sample of the original Markowitz model against the proposed model and against other state of the art shrinkage methods. It was found that ME (mean-entropy) models do not always outperform their MV (mean-variance) and robust counterparts, although presenting an edge in terms of portfolio diversity measures, especially for portfolio weight entropy. It further shows that when increasing return constraints on portfolio optimization, ME models were more stable overall, showing dampened responses in cumulative returns and Sharpe indexes in comparison to MV and robust methods, but concentrated their portfolios more rapidly as they were more evenly spread initially. Finally, the results suggest that it was also shown that, depending on the market, increasing return constraints may have positive or negative impacts on the out-of-sample performance.

## 1. Introduction

Capital markets have been one of the most popular vehicles of investing for individuals. They provide investors with real time information about security prices, liquidity and many opportunities for those who are seeking returns that are larger than the economy’s base interest rate, which is nowadays at historical all-time lows across the globe [1]. However, how does one invest their money? Considering a market with a diverse set of sectors companies and investments, which should be the ones to hold? What are the general rules that rational investors use to make their decisions?

Markowitz’s answer to these questions is in his seminal paper “Portfolio Selection” [2], which laid the foundations for Modern Portfolio Theory (MPT). There, he described how an investor could optimize his future returns given a certain amount of risk. He was later awarded the Nobel prize [3] for his contributions to finance, which he shared with William Sharpe for the creation of the Capital Asset Pricing Model (CAPM) [4] and Merton Muller, for his contributions on corporate finance. The revolutionary nature of his work was introducing variance, covariance and standard deviation as measures of risk, against which the future returns could be optimized.

Sharpe complemented Markowitz’s work by laying the foundations of CAPM, and by suggesting that the rational investor should hold a combination of the optimum market portfolio, i.e., the portfolio which had the highest Sharpe ratio (excess expected return/standard deviation), with the risk-free asset (a US treasury bill, for example). His suggestions are a byproduct of the addition of two assumptions to the MPT model: first, every investor sees the same price distribution, that is, there is complete agreement of the market regarding the portfolios located on the efficient frontier; and second, there is no additional costs for any investor when borrowing or lending money at the risk-free rate. In his original paper, Sharpe predicted that the optimum portfolio would lie within a region of feasible solutions (a line where the CAPM would slice the efficient portfolio sets), instead of being represented by a single point in the efficient frontier. That would be because, as the market concentrated their assets on the optimum point, the securities that were included in the portfolio would experience a price increase, diminishing the future expected returns, hence their Sharpe ratios, which would then be replaced by more moderately priced securities. That cycle would repeat itself continuously, as markets operate.

Though the combined works of Markowitz and Sharpe are considered to be the foundations of MPT, both its underlaying assumptions and empirical application have been object of criticism in literature. In practice, the resulting optimized portfolios tend to be concentrated and present low out-of-sample performance, comparable to naïvely diversified strategies, as proposed by [5]. More recently, a new way to better disperse the portfolio weights has been explored with the usage of entropy, both as a risk variable and a diversity constraint. Entropy can be interpreted as representing the uncertainty of a random variable and, in our particular case, it is used as an analogy to variance. Similarly, mutual information (MI) is a measure of dependence between two random variables and corresponds to covariance. Unlike variance and covariance, entropy and MI do not depend on the assumption that the underlaying distribution of the random variable is normal.

Portfolio optimization is a key business area surrounded by epistemic uncertainty with respect to the duo formed by the object under study and the chosen method to decide upon asset allocation. Epistemic uncertainty is lack of knowledge on underlying fundamentals or total ignorance of, for example, a possible alternative scenario. This epistemic uncertainty is inherent to the delimitation of the object–method pair under study and manifests itself regardless of the identified literature gap, the alternative models used, the stock market selection and the reproducibility conditions that are intrinsic, to some extent, to the idiosyncrasies of each country. While the proper identification of a literature gap is relevant for advancing the body of knowledge, especially in portfolio optimization models, where a plethora of methods are designed to treat specific aspects of how much of each asset should be allocated, research gaps do not themselves mitigate epistemic uncertainty, only assuring the aspects of internal validity—in light of the current body of knowledge—and scale validity—that is, if the proper analytical models were developed to adequately handle the specific nature of what is being measured. Hence, the choice of the key measurements for assessing epistemic uncertainty in stock markets is the relevant issue for assuring research reproducibility with respect to distinct countries, where generalization of results can be developed and compared with more certainty.

Hence, as regards this paper, epistemic uncertainty can be conceptualized as the scientific uncertainty in the process of modeling. It is due to limited data and knowledge, and possible tools to mitigate epistemic uncertainty in the field of soft computing are the execution of sensitivity analysis, not only by running alternative models, but also using different parameters into the proposed model; and the apprehension of information entropy principles, for improving decision making as regards whether or not a given model is contributing to reducing epistemic uncertainty knowledge. Information entropy is the cornerstone of information theory, providing a constructive criterion for setting up probability distributions of computed scores on the basis of partial knowledge, while enabling a type of statistical inference based on the heterogeneity or dispersion of the scores where no extra biases or uncalled assumptions can enter into the analysis.

Focusing on solving the problems regarding its application, the present work is dedicated to describing a new model for portfolio optimization (PO), using entropy and mutual information instead of variance and covariance as measurements of risk. These measures were introduced by [6] in his seminal paper “A Mathematical Theory of Communication”.

This work will also investigate which are the measures with better results in real world data. For this end, it will compare the performance in and out of the sample of the original Markowitz model against the proposed model and against other state of the art shrinkage methods of [7,8,9]. The performance measures utilized are the portfolio return, risk, risk adjusted return measures and Jensen’s alfa. The databases utilized are the constituents’ stocks of the DJIA, IBOVESPA and S&P500 indexes, with monthly and daily returns being separately tested between 2015 and 2019. Exhaustive tests were performed with MI and its many normalized variants, with the aim of selecting the best performer. We also test the results with varying sets of return constraints, to understand how the proposed method’s results are sensitive to changes in input parameters. Section 2 describes the Markowitz PO problem, Shannon’s entropy measures and present the literature review on the subject. Section 3 is dedicated to the methodology, Section 4 to results and Section 5 concludes.

## 2. Literature Review

- Introduction to Markowitz’s model

Let *m*(*n*,*t*) represent the matrix containing the realization of prices *p* of assets 1 to *n* within its columns, and for times 1 to *t* within its rows, as shown in Equation (1). The relative price change $p{\left(i\right)}_{t}/p{\left(i\right)}_{t-1}-1$ represents the period return of such prices. This calculation can be applied to each column (security) of matrix *m* to achieve the return matrix *k*(*n*,*t*), represented by Equation (2), which is used as the main input for PO problems. Originally, [2] described the PO model as a quadratic programming problem, minimizing risk restricted to the minimum desired portfolio return, $r_{min}$, as in Equation (3). In his formulation, he also included short-sale restrictions and equalized the sum of all weights to 1. It is also important to specify that, in this case, variance and covariance were the measures utilized to represent risk, with ∑ representing the *n* by *n* covariance matrix of Equation (2) while the portfolio return is the 1 by *n* column vector denoted by $\bar{p}$ containing the average returns of Equation (2). The optimization variable is the portfolio weight vector $x_{n}$, which holds the information of the proportion invested in assets 1 to *n*.

(1) $$m\left(n,t\right)=\left|\begin{array}{cccc} p{\left(1\right)}_{1} & p{\left(2\right)}_{1} & \ldots & p{\left(n\right)}_{1}\\ p{\left(1\right)}_{2} & p{\left(2\right)}_{2} & \ldots & p{\left(n\right)}_{2}\\ \ldots & \ldots & \ldots & \ldots \\ p{\left(1\right)}_{t} & p{\left(2\right)}_{t} & \ldots & p{\left(n\right)}_{t} \end{array}\right|$$

(2) $$k\left(n,t\right)=\left|\begin{array}{ccc} p{\left(1\right)}_{2}/p{\left(1\right)}_{1}-1 & \ldots & p{\left(n\right)}_{2}/p{\left(n\right)}_{1}-1\\ p{\left(1\right)}_{3}/p{\left(1\right)}_{2}-1 & \ldots & p{\left(n\right)}_{3}/p{\left(n\right)}_{2}-1\\ \ldots & \ldots & \ldots \\ p{\left(1\right)}_{t}/p{\left(1\right)}_{t-1}-1 & \ldots & p{\left(n\right)}_{t}/p{\left(n\right)}_{t-1}-1 \end{array}\right|$$

(3) $$\begin{array}{c} minimize\;x^{T}\sum x\\ subject\;to\;{\bar{p}}^{T}x\;\geq r_{min}\\ \;\;\;\;\;\;\;1^{T}x=1,\;x\geq 0 \end{array}$$

There are also some variations in the original problem found in the literature, such as the bi-criterion problem presented in Boyd and Vandenberghe’s book [10], represented by Equation (4), where the return constraint subtracts (hence, it is maximized) from the covariance matrix in the objective function and a new constant µ, which multiplies the covariance matrix, represents a risk aversion parameter. This version is particularly useful for the numerical calculation of the efficient frontier: by varying µ, we get a range of Pareto efficient portfolios from the minimum variance (when µ is large) to maximum return (when µ is small).

(4) $$\begin{array}{c} minimize-{\bar{p}}^{T}x+µx^{T}\sum x\\ subject\;to\;1^{T}x=1,\;x\geq 0 \end{array}$$

Another noteworthy formulation is Sharpe’s definition of the market portfolio [4], which is an integral part of his CAPM theory. In this version, the problem consists of the maximization of the Sharpe ratio, i.e., the portfolio with the highest risk adjusted return in the efficient frontier, as demonstrated in Equation (5). According to CAPM theory, a combination of this portfolio with the risk-free asset (lending of borrowing money at the risk-free rate, with no added costs) should always be held by all, so called, rational investors, because it would represent the best tradeoff between risk and return. This would be an inevitable conclusion if the premises of Sharpe and Lintner, i.e., complete agreement of observed price distribution by the market and borrowing and lending at risk-free rate, were true.

(5) maximize x−rfxT∑xsubject to p¯Tx ≥rmin       1Tx=1, x≥0

- Criticism in literature

Though, the empirical application of mean-variance models is met with some challenges. The most common drawback cited in the literature is the rather theoretical observation that the distribution of the return series is not normal, hence mean and variance lose their maximum likelihood estimator status and would not be ideal for parameter estimation. A greater susceptibility to errors is expected then, and its implications explored in [11], who find that the model is 10 times more sensitive to errors in the mean vector than in the variance–covariance matrix. They conclude that setting the expected returns vector to zero can lead to a “better portfolio allocation”, and that investors who are less risk averse should invest more time into estimating future returns. There are other solutions proposed in the literature, such as robust optimization [12], the addition of higher moments (skewness and kurtosis) to the objective function [13] or changing the risk metric altogether [14]. On the last issue, there is a substantial body of literature dealing with linearization of the mean-variance model, making it simpler to be solved, by adopting semi-variance measures such as CVaR (conditional value at risk) or MAD (mean absolute deviation) [15] metrics instead of variance. Although relatively less explored than these alternatives, entropy has also made a resurgence in recent literature, and will be further investigated later.

The lack of real-life constraints on the original model, such as short-sale (SS), boundary constraints (BC), cardinality (CC), fixed, variable and transaction costs (TC), transaction lots (TL), sector capitalization (SC) and turnover (TU), is also a concern debated in the literature. SS restricts the portfolio to holding only long positions; BC imposes bounds on the value of each asset weight; CC imposes bounds on the number of invested securities; TC is the fee investors pay when buying or selling securities; TL ensures the amount invested is a multiple of the minimum transaction lot; SC ensures that sectors which are more capitalized have higher weights; and TU sets the turnover rate from one period to the next, especially useful in multi-period PO. As pointed out in [12,15,16], the addition of some of these constraints in the model may render it non-convex or NP-hard, which in turn favors the usage of heuristic algorithms or mixed integer linear programming (MILP) methods for solution search instead of quadratic programming (QP) techniques. In the present work, only short-sale constraints will be utilized in the model in conjunction with QP. For a comprehensive taxonomy of optimization algorithms used in portfolio optimization see [16], who reviewed and classified 175 PO works and their respective solution methods. Ref. [15] focused on MILP techniques, while ref. [17] focused on documenting exact and heuristic methods, software/programming languages, constraints, and types of analysis (technical and fundamental) regarding PO.

Another area for improvement is changing the model from single period to multi-period optimization. This solution focuses on relaxing the restrictions of single period models, i.e., that investors can only rebalance their portfolios at the end of each investment period. Dynamic models make this process run continuously and have been developed both in discrete and continuous time intervals. In this case, dynamic programming and stochastic techniques are used to solve the problem instead of QP. In the present work, we will be using a single period model, and leave the multi period application for further studies. For further reading regarding this subject, see [12].

As for out-of-sample performance, ref. [5] have shown that naïve diversified portfolios (equally distributed weights over *n* assets, 1/*n*) have better performance than popular mean-variance models including classical, Bayesian approach to estimation error, moment restrictions and with portfolio constraints. Their simulations show that these models should only beat naïve portfolios when the number of securities is small compared to the periods of estimation, and when “very high” levels of idiosyncratic volatility are present. They also find that short-sale constrained models (holding only long positions) perform better than unconstrained, and that minimum variance portfolios perform better than their alternatives in terms of out-of-sample risk adjusted performance. In fact, they highlight this result while citing the work of [18], which also found that “the sample covariance matrix performs almost as well as those constructed using factor models, shrinkage estimators or daily returns”. These findings are in consonance with Chopra and Ziembra [11] reported earlier. Based on this knowledge and aiming towards the small, unsophisticated investor, Leal and Campani [19] proposed a minimum variance index fund for the Brazilian market, composed of 20 stocks of the Ibovespa index. 

Regarding CAPM, one of the most comprehensive critiques came from Fama and French [20], who analyze the differences observed between theory predictions and empirical tests’ results. They found out that, even in its relaxed form proposed by [21], the main conclusions of the theory did not hold in practice. In this case, market betas do not reflect all available information and other extrinsic variables, such as stock price ratios of book to market value and price to earnings, have information about expected returns missed by market betas. As CAPM partly stems from the same assumptions utilized in Markowitz modern portfolio theory (MPT), i.e., that investors only care about the mean and variance of their returns, the practical refutation of CAPM also puts into question these foundations. In Fama and French’s own words: “If betas do not suffice to explain expected returns, the market portfolio is not efficient, and the CAPM is dead in its tracks.” and indeed, also MPT and the mean-variance model, which they call a “theoretical tour de force”. According to them, these models serve a didactic purpose, but they need to be enhanced to ensure practical use. More recently Fama and French proposed the five-factor model [22], which includes other variables in the original CAPM to explain observed empirical anomalies.

Although factor models are a potential way of redeeming CAPM and MPT from estimation error, other authors are not convinced of their universal applicability regarding PO and risk mitigation. Ref. [7] argue that choosing between factor models is very dependent on the circumstances and many times one must look out-of-sample to verify and adjust one’s model accordingly. Therefore, one can never be sure about the success of a factor model a priori. Instead, they suggest other ways to deal with errors in parameter estimation by utilizing matrix shrinkage techniques [7,8,9], representing one of the robust optimization techniques available, regarded by some as state of the art [23]. These models will be tested against the proposed model in this paper.

Finally, another promising research area is the combination of fuzzy logic with PO, that aims to imbue the model with the uncertainty present in future expected returns, hence reducing parameter estimation errors. This is especially useful to include financial analysts’ beliefs on the likelihood of future events and other variables, making the model account for these effects during the optimization process [24]. Although the literature is vast, according to [12], few articles focus on real life applicability of this tool, making it a good candidate for future research. The present work is focused on using the standard probabilistic model and will leave the incorporation of fuzzy variables for future studies.

- Introduction to entropy

The term entropy was originally applied in physics, first by Rudolph Clausius, who introduced the concept as meaning disaggregation in the microscopic structure. Later, Ludwig Boltzmann generalized the concept and interpreted it as the number of alternative microscopic configurations or states of individual atoms and molecules in a system. By doing so, he brought the concept of statistical disorder and probability distributions into statistical mechanics. His definitions are central to the second law of thermodynamics.

In 1948, Claude Shannon founded the branch of information theory with its seminal paper “A mathematical theory of communication” [6], in which he applied Boltzmann’s formulations to describe the loss of information in telecom signals. In this case, entropy represents the minimum descriptive complexity of a random variable, or its average uncertainty. He also introduced mutual information as a measure of communication rate between signals in the presence of noise and it represents the reduction in uncertainty of one random variable due to the realizations of another. In other words, mutual information I (*X*; *Y*) is a measure of the dependence between the two random variables. It is symmetric in *X* and *Y*, always nonnegative and is equal to zero if and only if *X* and *Y* are independent [25].

- Mathematical definitions

The entropy of a random variable *X* with a probability mass function *p*(*x*) is defined by [25]:

(6) $$H\left(X\right)=-\underset{x}{\sum }p\left(x\right)log_{2}p\left(x\right)$$

In this work, logarithms to base 2 are used, hence entropy can be interpreted as the number in bits required to describe a random variable, in this case, the series of historical returns.

To define mutual information, consider two random variables *X* and *Y* with a joint probability mass function *p*(*x*, *y*) and marginal probability mass functions *p*(*x*) and *p*(*y*). The mutual information I (*X*; *Y*) is the relative entropy between the joint distribution and the product distribution *p*(*x*)*p*(*y*) [25]:

(7) $$I\left(X;Y\right)=\underset{x\in X}{\sum }\underset{y\in Y}{\sum }p\left(x,y\right)log\frac{p\left(x,y\right)}{p\left(x\right)p\left(y\right)}$$

This definition can also be written as:

(8) $$I\left(X,Y\right)=\underset{x,y}{\sum }p\left(x,y\right)log\frac{p\left(x,y\right)}{p\left(x\right)p\left(y\right)}$$

- Entropy usage in finance and PO problems

Entropy and mutual information have been extensively applied in financial literature. Ref. [26] reviewed the applications of entropy in finance and highlighted its usage in portfolio optimization and asset pricing. The motivations and expected results from both applications are different. In the first case, entropy is used as an alternative to variance because it does not require any prior knowledge regarding the underlaying distribution, as opposed to variance that only retains MLE status under normality, and because of its presumed greater generality while dealing with higher moments and non-linear relationships between data. These features (non-normality, non-linearity of dependencies and presence of higher moments) are known to be present in financial series, so entropy is a prime candidate to remedy the theoretical drawbacks of classical mean-variance models. Though no conclusion can be made on how this modification will affect the result a priori, Ref. [27] have shown that optimal mean-entropy portfolios are also mean-variance optimal and vice versa. As in the mean-variance model, entropy is minimized, because the goal is to reduce uncertainty of returns. In the second case, entropy is used in conjunction with other variables, typically variance, higher moments such as skewness or kurtosis, or semi-variance, or a combination of these, which represent the risk measure to be optimized against. It can be part of the objective function on multi-objective problems, or it can be handled as a constraint. In either case, *p*(*x*) is substituted with the weights of the portfolio, instead of representing the realizations of historical price returns. It is clear to see that when one holds the 1/*n* portfolio, maximum entropy n is achieved, while if the output portfolio is concentrated on only one asset, the entropy is equal to 1. Hence, instead of minimizing, the goal is to maximize entropy, with the clear expectation that the resulting portfolio will be more disperse than the standard benchmark model, whatever it may be. As negative numbers do not have logarithms, short-sale constraints are automatically applied, unless otherwise stated.

Compared to entropy, mutual information has seen limited use in finance, but recent literature successfully applied it in many areas. The first successful use case found was to measure dependencies between lagged time series in [28], which then served as a basis for the work of [29] that used entropy and MI as measures of dependency in financial series. Ref. [30] also applied MI to measure dependencies for non-linear timeseries and [31] introduced MI and MI rate to incorporate non-linear dynamics. Ref. [32] then analyzed the relationship between global and linear correlations of financial returns and found that the global coefficient provided by MI was more general and capable of revealing dependencies between security returns. Ref. [33] analyzed information sharing between different markets before and during the COVID-19 pandemic and concluded that MI in conjunction with linear correlation can be used as a potential early warning indicator for market crashes. Ref. [34] also applied MI and the global correlation coefficient to measure operational risk aggregation in Chinese banks, finding that the generality of MI could also be useful in this case. By their calculations, they estimated that banks could almost half their provisions considering the available risks at that time. There is no application of this measure in studies regarding PO models, to the best knowledge of the author. It should be noted though that Ref. [35] propose a PO model that minimizes the cross entropy between the optimized portfolio and a benchmark, suggesting the 1/*n* portfolio as a candidate to ensure diverse output weights. Mutual information can be interpreted as a particular case of cross entropy, but in this study the measure is used as a shrinkage target to the optimal portfolio, and not to calculate the dependencies between securities and their historical returns, as the present work suggests.

- Entropy usage as a risk measure

Regarding entropy as a risk measure, Ref. [27] were the first to propose the mean-entropy model. There, they suggested the use of joint entropy between the securities and a market benchmark as a measure of risk, to reduce computational complexity of having to calculate joint entropies for each pair of stocks. They show that mean-variance efficient portfolios are also mean-entropy efficient, and conversely. Ref. [36] systematically test six variations of mean-entropy models (using derivations of entropy measures such as Information Entropy and Cumulative Residual Entropy in the probability space, Fuzzy Entropy, Credibility Entropy and Sine Entropy in the fuzzy space, and Hybrid Entropy in the hybridized uncertainty of both fuzziness and randomness) against the standard mean-variance approach. They analyze each of the variations with respect to the mathematical properties of “effective risk measures”, i.e., monotonicity, Translation Invariance, Sub-additivity, Positive Homogeneity, Consistency and Convexity. They conclude that none of the proposed measures pass on all the six theoretical tests. They also analyze the models with respect to cumulative returns on both Chinese and American stock markets, resulting in the fuzzy entropy model (MFEM) outperforming mean-variance on both cases out-of-sample. Other models are more successful than mean-variance on the Chinese case, while only MFEM can out-perform it on the American dataset.

Ref. [24] propose a fuzzy mean-entropy model to accommodate the non-normality of returns and optimize portfolios regardless of symmetric requirements on membership functions of fuzzy returns. They compare it with fuzzy mean-variance models in a simulated case with 10 securities and use hybrid intelligent algorithms instead of QP. Ref. [35] use cross entropy objective function to shrink the optimized portfolio to the 1/*n* equally weighted portfolio. They test their model with different shrinkage intensities against six benchmark models and use Sharpe ratios and CEQ (certainty equivalent return) as comparable performance measures.

More recently, Refs. [23,37] proposed entropy and Rényi entropy as risk variables for the objective function, respectively. The innovation in [37] was to compute the probability mass function of entropy as the objective function, as opposed to calculating entropy prior to optimization, while Ref. [23] show that Rényi entropy features a parameter that can be tuned to play around with the notion of uncertainty, thus being able to adjust between the model being more sensitive to central or tail tendencies. Both show their proposed models to outperform the standard mean-variance model, while Lassance specifically tests against and outperforms other robust MV estimators.

- Entropy usage as a diversity measure

Regarding entropy as a diversity measure, Ref. [38] compares it against other popular diversity constraints regarding their ability to shrink the feasible region of the PO problem. He shows that, by transforming these constraints into a canonical form and using common upper bounds, they exhibit a subset relation among their feasible solution, concluding that entropy is the least restrictive amongst the studied methods. 

Ref. [39] systematically test three different entropy measures (entropy, Yager’s entropy and minimax disparity, which is a linear entropy model), with varying sets of constraints (short-sale restricted or not) and objectives (standard mv, min variance, minimize transaction costs). They conclude that the addition of short-sale constraints reduces the out-of-sample performance, in contrast to what earlier literature suggests [5,8,11], and that “the addition of portfolio objectives and entropies increases the mean–variance efficiency”. These tests were conducted with 136 securities within the TAIEX exchange, representing more than 80% of its capitalization.

Ref. [14] combine multi-period dynamical PO with fuzzy lower semi-variance, returns and entropy to introduce a possibilistic mean–semivariance–entropy model, also including transaction costs. The fuzzy approach enhances entropy of portfolio weights as a measure of diversification by introducing possibilistic entropy, which is a function of security weights multiplied by the risk adjusted performance of each respective security. If the security expected return is inferior to the risk-free asset, the weight of the particular security is defaulted to zero in the resulting optimization. As with many fuzzy proposed models, they tested it on simulated data for four stocks, considering only the return as a performance measure.

Ref. [40] propose a cardinality constrained MV model with entropy as a diversification measure. They also develop a modified genetic algorithm to solve the problem. They only test and record performance measures for the optimization algorithm execution. They show that the inclusion of entropy in the model enhances the algorithm’s performance in error metrics, since it betters diversifies the results, although it increases its execution time slightly. They test on various datasets, with a weekly return series. 

Ref. [41] apply entropy in conjunction with a mean-variance-skewness model (MVSEM). They argue that the addition of skewness to the mv model accounts for non-normality of series returns, but they acknowledge that it may concentrate portfolios. To remedy this, they include entropy as a diversity measure. They test the model with three different datasets of monthly returns and compare the portfolio performance with respect to Sharpe ratios, ASR, MADR, SSR, FTR and GRR measures. They conclude that the performance of the MVSEM is better than the benchmark models in many measures, while observing less portfolio turnover, hence reduced operational costs.

More recently, Ref. [42] proposed a self-adapting MVPO with entropy portfolio weights model. The self-adapting constant adjusts to market conditions, concentrating or diversifying portfolios accordingly. They test it with a five year daily returns series of the SSE50 index constituents and find that the proposed model helps to diversify the output portfolio and mitigate unsystematic risks, as initially intended. They use Sharpe index, max drawdown, Calmar ratio and win ratio as performance measures to compare against various benchmarks.

## 3. Model Description

- Proposed model description

As reported earlier in this work, there is a gap in the literature regarding the use of mutual information, a more general measure of dependency between variables, applied to PO problems. The usage of entropy as a risk measure is also less explored and most of the work has been directed towards treating it as a side constraint or as an additional goal in multi objective PO models. Having this in mind, we propose a model where variance and covariance are substituted by entropy and mutual information in lieu of the covariance matrix. In this case, entropy would serve as an analogy to variance and would be placed at the diagonal of the matrix, while MI takes the place of covariance in the matrix’s upper and lower triangles. By doing this, we aim to alleviate the problems of parameter estimation due to the non-normality of the return series, while at the same time accounting for non-linear relationships between them. Then, the problem can be defined as standard Markowitz and Sharpe models, as shown previously in Equations (3) and (5), respectively, while the sigma matrix takes a different form, where *e_n_* represents securities’ entropies and *mi_n_* their respective pairwise mutual information:

(9) $$\sum =\left[\begin{array}{c} \begin{array}{ccc} e_{1} & mi_{21} & mi_{2n}\\ mi_{12} & e_{2} & \ldots \\ mi_{1n} & \ldots & e_{n} \end{array} \end{array}\right]$$

We also test different normalized variants of MI in the triangles of the matrix, which are of the form:

(10) $$MI\left(X;Y\right)=\frac{I\left(X;Y\right)}{C_{n}}$$

where *C* can assume the following values:

(11) $$\begin{array}{c} C_{X+Y}=H\left(X\right)+H\left(Y\right)\\ C_{MIN}=\min\left\{H\left(X\right),H\left(Y\right)\right\}\\ C_{MAX}=\max\left\{H\left(X\right),H\left(Y\right)\right\}\\ C_{XY}=H\left(X,Y\right)\\ C_{SQRT}=\sqrt{H\left(X\right)H\left(Y\right)} \end{array}$$

The substitution of variance and covariance in the matrix with entropy and mutual information may cause unwanted effects on the resulting equations, as variance and covariance units are expressed in squared returns, which is itself unitless, while entropy and mutual information are expressed in bits, since this work utilizes logarithms of base 2. While the mathematical soundness of the model needs to be further investigated, it falls outside the scope of this work, which focuses on the systematic analysis of the proposed model’s results.

At this point, it is important to mention that during the discretization process of the series of returns, an interval must be chosen to count the various realizations of the variable. In the present research, 101 different states were considered, between −50% and +50% return with 1% increment steps. As Ref. [37] point out, “the selection of intervals is at the discretion of the user” while suggesting that “the user should explore reasonably sized intervals that yield the intended level of risk mitigation across portfolios”. It should also be mentioned that any discretization process may introduce some kind of bias during the estimation of entropy and MI. For more details on density estimation, see [37].

It is also reasonable to assume that the proposed models will not outperform regular MV portfolios on every possible scenario. Having this in mind, an optimization routine where an algorithm searches for the best possible returns given the portfolio allocations suggested by each method was also implemented, being able to freely distribute weights between these solutions. By doing this, we hope to achieve a more robust solution to PO. This model shall be listed in test results as “Combined”, since it originates from the combination of previous optimized portfolios. For this purpose, we choose the heuristic differential evolution technique proposed by [43], since the objective function is possibly non-linear and non-differentiable, rending quadratic solvers unsuitable to find a global solution.

For each resampled covariance matrix of B3, DOW, SP stock market returns, the average eigenvalue ratios were, respectively, 638.98, 89.06 and 33,093. Such values denote the condition number for the samples for each stock market, measuring how much the output value of the function—expected return of the portfolio—can change for a small change in the input argument—or a given stock within the portfolio. A PO problem with a low condition number is said to be well-conditioned, while a problem with a high condition number is said to be ill-conditioned. One can easily note that the condition numbers for each one of the three stock markets under analysis is quite heterogeneous. Hence, and in line with [5], we deemed it necessary to utilize robust estimators of the covariance matrix, as in [7,8,9]. These models are robust shrinkage estimators that minimize the Frobenius norm, denoted by ∂, between the shrinkage estimators and the sample covariance matrix ∑. These three target matrices are denoted by $F_{CC}$, a constant correlation model, $F_{SF}$, a single-factor model and $F_{I}$, a scalar multiple of the identity matrix. These will be applied to enhance the performance of the standard covariance matrix in the mean-variance approach.

(12) $$\sum_{CC}=\partial F_{CC}+\left(1+\partial \right)\sum \;$$

(13) $$\sum_{SF}=\partial F_{SF}+\left(1+\partial \right)\sum$$

(14) $$\sum_{I}=\partial F_{I}+\left(1+\partial \right)\sum$$

- Optimization algorithms

As the present work maintains the same structure of the original mean-variance model, it assumes that the resulting optimizing function remains convex, even though entropy has been demonstrated to be concave [25]. Hence, the optimization models utilized are quadratic programming tools, which are perfectly suited to these kinds of problems and guarantee convergence to the global minimum under convexity, designed for python and publicly available at CVXOPT and scipy.minimize (using SQSLP method), which is a well-known machine learning library. The first source uses cone programming and interior point methods described by [44] to find the solution, while the second implements sequential quadratic programming methods as defined by [45].

For the calculation of the efficient frontier, CVXOPT is utilized, while SQSLP method is used for the calculation of PO models, as it provides a more convenient way to handle constraints. In our empirical tests, all SQSLP generated optimal portfolios were on the efficient frontier set by CVXOPT. For more information on these kinds of algorithms, we refer the reader to [46]. To compute the combination of optimized portfolios, we use differential evolution techniques also publicly available in scipy package (scipy.optimize.differential_evolution). It implements the algorithm described by [43], as pointed out earlier, its solution is also short-sale restricted.

- Empirical analysis

To ensure that the proposed model has practical use, it is submitted through a series of backtests regarding different return measurement intervals (daily or monthly), increasing constraints on returns, from minimum variance or entropy until max expected return portfolios, and different stock indexes from the USA and Brazil (Dow Jones, S&P500 and Ibovespa), to verify the empirical results where different levels of idiosyncratic volatility are observed (generally speaking, DJ < S&P < IBOV). A time period of 5 years is analyzed, between January 2015 and December 2019, with a varying number of securities n, depending on the index. Stocks with more than 100 days of missing data between this time interval are excluded from the analysis, so n = 29 for DJIA, 440 for S&P500 and 66 for IBOV. We chose to exclude 2020 because of the added market volatility due to the COVID-19 pandemic. The benchmark models have proven to be robust in past literature and are the equally weighted portfolio, the mean-variance model and its robust shrinkage estimators variants proposed by [7,8,9].

To measure and compare performance between different models, we select popular measures of risk adjusted returns, excess returns, and overall risk. The Sharpe index measures the risk adjusted returns regarding the mean and standard deviation of returns, as defined by [4]:

(15) $$Sharpe\;Ratio=\frac{E\left[return\right]}{\sqrt{\sigma^{2}\left[return\right]}}$$

To measure risk, we consider standard deviation and the p1 and p99 percentiles representing the range of abnormal losses and returns.

To measure consistent risk adjusted abnormal returns, we make use of the Jensen’s measure comparing each model against the mean-variance approach, as defined by [47]:

(16) $$\alpha =r_{i}-\left[r_{f}+\beta \left(r_{m}-r_{f}\right)\right]$$

where $r_{i}$ represents the realized return of the portfolio or investment; $r_{m}$ the realized return of the market benchmark, in the case of this work represented by mean-variance portfolio; $r_{f}$ the risk-free rate of return for the period and β is the beta of the portfolio of investment with respect to the MV portfolio.

This work also measures portfolios according to the diversity sof output weights, to identify if the proposed models better disperse their risks as compared to the benchmark. As reported earlier in this study, entropy has become a popular measure of diversity applied in PO literature, hence, we implement it here as, where $w_{k}$ represents the weight of the asset *k*:

(17) $$Portfolio\;Entropy=e^{-\sum_{k=1}^{N}w_{k}\ln\left(w_{k}\right)}$$

Though entropy has risen in popularity as a diversity proxy, [48] classify it as an “effective number of bets” measure, meaning that it is not very different from just counting the number of assets invested in the portfolio. This type of measure suffers from two drawbacks: first, when applied to portfolios containing non-homogenous risk assets, one might wrongly assume that the risks are diversified away, while if one invested asset carries naturally more risk this may not be the case; and second, it can also be deceiving when applied to assets with correlated risks, creating the impression that the portfolio is diversified while this configuration may increase portfolio volatility.

To account for correlation adjusted diversity and following the [48] suggestions, the measure proposed by [49] is also included. It can be defined as:

(18) $$GLR\left(w\right)=\frac{w^{T}\sum w}{\sum_{k=1}^{N}w_{k}\sigma_{k}^{2}}$$

This metric considers not just the quantity of invested assets, but also their correlation. A portfolio with a high concentration of weights in assets with strong correlation will have a portfolio risk that is higher than the average standalone risk of each of its members. As a result, it will have a high GLR.

## 4. Results

In this section, we present the results from the backtests performed on DJIA, Ibovespa and S&P500 index constituents. For each one, the results of all the models tested are compared considering all the performance measures mentioned previously and different measurement intervals (daily or monthly prices). Table 1 contains the abbreviations utilized for each model for data labels. Finally, the similarities and differences between each index’s backtests results are discussed. The resulting weight distribution for the combined portfolio will be included in the annexes for each run.

- DJIA

For the daily returns of 29 DJI’s constituent stocks from January 2015 to December 2019, with 839 days being used to train the model and 419 for testing the output weights while imposing no return constraints (in that case, minimizing variance and entropy), the results are shown in Table 1.

Table 2 reports the DJIA daily return test. For this case, MV and robust models dominate ME in terms of return, risk and risk adjusted return measures, with all alphas being negative except in Ledoit Wolf’s single factor model. In terms of portfolio diversity though, ME models tend to spread the portfolio’s weight more evenly than mean-variance, which concentrate the portfolio into 8–10 assets. In terms of correlation adjusted diversity, ME models also perform better than their MV counterparts, resulting in more conservative portfolios, even though the observed volatility during the period is slightly higher than that shown in the MV models. The only model that returned a positive Jensen’s measure in relation to standard mean-variance was Ledoit Wolf single market factor model, which also presented better abnormal returns (P99) and Sharpe index while also slightly increasing portfolio concentration. All maximum Sharpe models presented worse results than their minimum variance/entropy counterparts, while only two were beaten by the naïve portfolio (combined and MV Sharpe). The combined and MV Sharpe portfolios share very similar results, as the optimization algorithm could not find better solutions, resulting in the combined portfolio holding almost all its assets in the best in sample performer, in this case, the MV Sharpe.

Now, considering monthly returns instead of daily returns, with 40 months being used to train the model and 20 to test while also imposing no return constraint, the following results were achieved (Table 3):

Table 3 reports the DJIA monthly return test. In this scenario, MV and robust models continue to present better returns, volatility and risk adjusted returns than ME models, though in this case, they also show better correlation adjusted diversity. All MV and robust models were able to outperform the naïvely diversified portfolios, while only the maximum Sharpe ME model had that capacity amongst ME variants. It is important to note though that the number of invested securities had only small changes from one test to another, demonstrating that normalized ME portfolios still more dispersedly distribute the weights amongst securities than MV. An exception for this rule is the non-normalized ME model, which has shown a relevant increase in terms of portfolio concentration from daily to monthly returns tests, while also performing poorly in general. The combined portfolio still resulted in a high concentration of assets with MV Sharpe model.

Lastly, the first test (for daily returns) is then rerun with constraints of 10%, 20% and 30% minimum return within the sample. For this test, a different approach is taken for result visualization: instead of showing a single table for every test, the analyzed performance indicator is compared for all models in a chart containing all tests to understand its evolution for each increment in the return constraint. The indicators analyzed are cumulative return, Sharpe index, portfolio entropy and the GLR measure.

**Figure 1:**
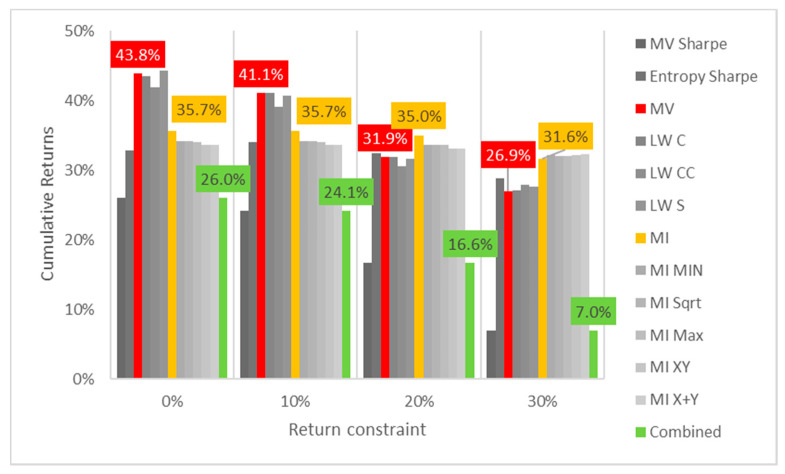
Cumulative returns x return constraint for daily DJIA return series; series appear in the same sequence of the data labels. Colored series denote MV, ME and combined models.

Figure 1 shows the culmulative returns x return constraint for daily DJIA return series. It is curious to see that raising the return constraint has the opposite effect on the out-of-sample performance, for each increment in required return, the cumulative output return decreases, at least in this case. MV and robust models show greater sensitivity and steeper declines than their ME counterparts. Sharpe variants of both MV and ME models also are more sensitive to an increase in return constraints than their standard counterparts.

**Figure 2:**
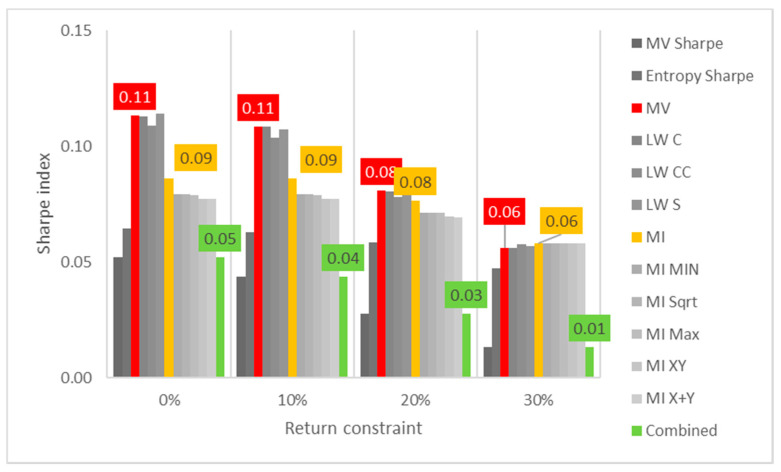
Sharpe indexes x return constraint for daily DJIA return series; series appear in the same sequence of the data labels. Colored series denote MV, ME and combined models.

Regarding Sharpe indexes, the same effect is noticed, as reflected in Figure 2, where MV and robust models appear to be more sensitive than ME models. In this case, it is also relevant to point out that Sharpe indexes are decreasing for each increment on the return constraint, showing that trying to establish a minimum output return might negatively affect the output portfolio not only in terms of reduced returns, but also more volatility.

**Figure 3:**
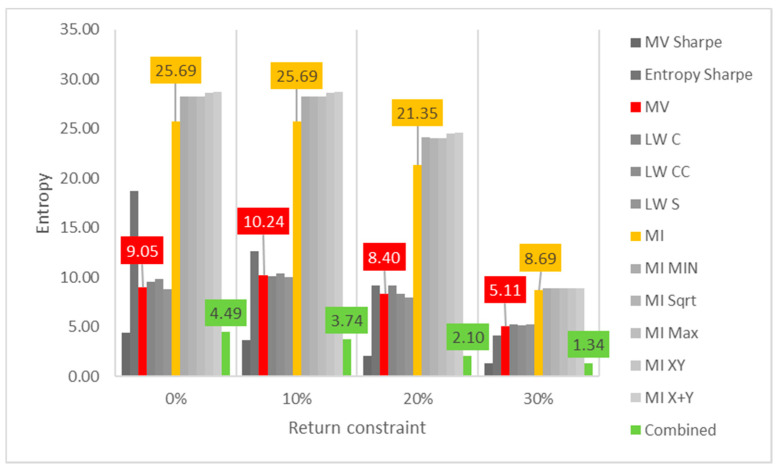
Entropy x return constraint for daily DJIA return series; series appear in the same sequence of the data labels. Colored series denote MV, ME and combined models.

Figure 3 reports the entropy x return constraint for daily DJIA return series. The same disparity in sensitivity to return constraint increases is also observed in diversity measures. In this case, however, ME models show steeper declines in portfolio entropy than MV and robust ones, maybe because they start much higher when compared to those.

**Figure 4:**
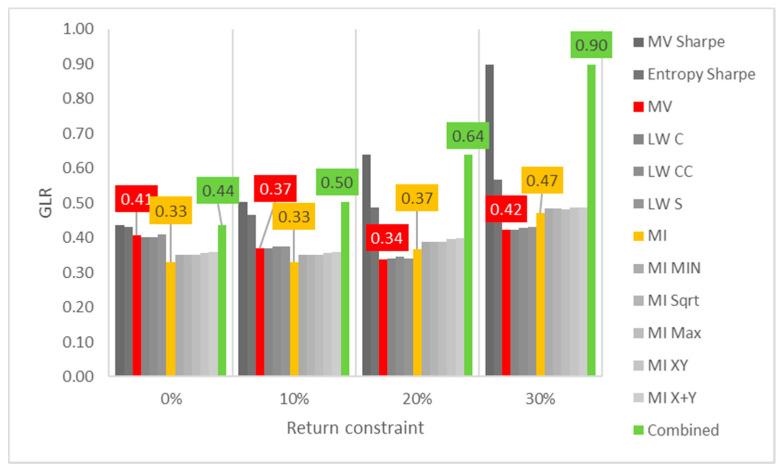
Entropy x return constraint for daily DJIA return series; series appear in the same sequence of the data labels. Colored series denote MV, ME and combined models.

Figure 4 shows the entropy x return constraint for daily DJIA return series. In terms of GLR, MV and robust models appear to be able to reduce correlation adjusted concentration, despite the decrease in number of assets held in the portfolio (lower entropy). However, as the return constraints become more restrictive (at 30%), even these models increase GLR. ME models on the other hand, present an increasingly upward trend of correlation adjusted concentration, matched with the aforementioned step decline in portfolio entropy.

- Ibovespa

Considering the daily returns of 66 Ibovespa’s constituent stocks from January 2015 to December 2019, with 827 days being used to train the model and 417 for testing the output weights while imposing no return constraints (in that case, minimizing variance and entropy), the results are reported in Table 4 as follows:

The only model capable of outperforming naïve diversification in this case was the Sharpe variation of ME, while all others fall short in out-of-sample performance, especially MV and robust models. At the same time, Sharpe variants of both ME and MV models outperform their standard counterparts. These results are the opposite of what was observed in earlier tests with DJIA stocks. Regarding diversity measures, ME models have higher portfolio entropies than MV and robust ones, however, they have similar correlation adjusted concentration, showing that MV and robust models reduce systematic risks more efficiently than ME in this case. The combined model continues to allocate the majority of its capital in the MV Sharpe model.

Now, considering monthly returns where 40 months are used to train the model and 20 to test while also imposing no return constraint, the following results in Table 5 were achieved:

The results in terms of performance measures are similar to what were shown for the previous test with daily return series, but in this case every model had reduced cumulative returns except Sharpe variant of ME and robust models. However, in terms of diversity measures the results are slightly different. In this case, ME and robust (except LW CC) models do better than MV ones in both portfolio entropy and correlation adjusted concentration, whereas in the previous test they had equivalent GLR measures. This is also the first test where the combined portfolio was able to find a more disperse solution, distributing asset weights all of the 13 models.

Finally, the models’ responses to change in the return constraint were analyzed using the same method as the DJIA case, that is, the daily returns series tests were rerun, however, the constraint increments were increased from 10% to 30% each, since the minimum variance/entropy returns are already much higher than DJIA so, instead of 10%, 20% and 30% minimum return, 30%, 60% and 90% were used.

**Figure 5:**
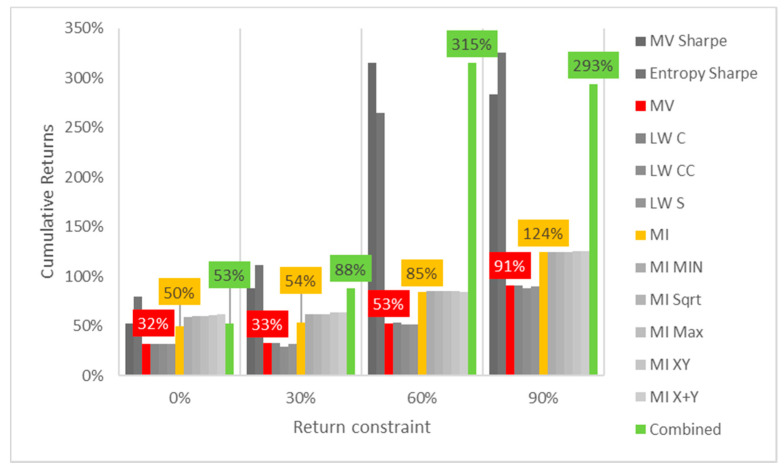
Cumulative returns x return constraint for daily Bovespa return series; series appear in the same sequence of the data labels. Colored series denote MV, ME and combined models.

**Figure 6:**
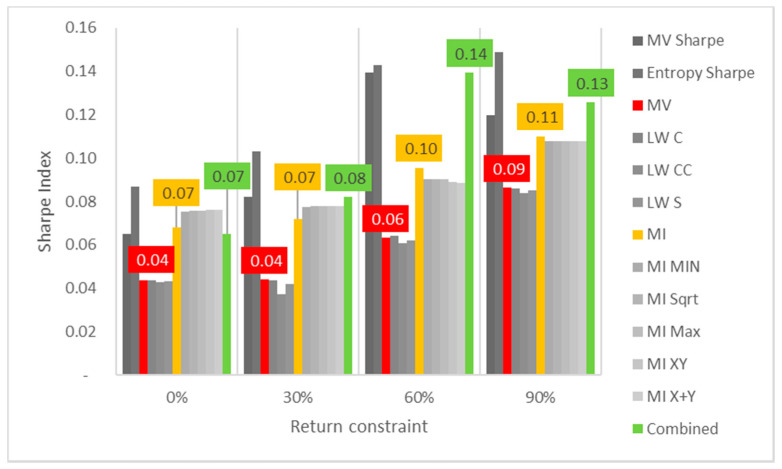
Sharpe indexes x return constraint for daily Bovespa return series; series appear in the same sequence of the data labels. Colored series denote MV, ME and combined models.

In contrast to what was observed in the DJIA case, where cumulative returns and Sharpe indexes would decrease for every increment in the return constraint, here they increase slightly for standard ME, MV and robust models, as observed in Figure 5 and Figure 6. The rate of increase is higher in the Sharpe variants of both models, but MV Sharpe experiences a slight decrease in returns and Sharpe at the last increment in the return constraint.

**Figure 7:**
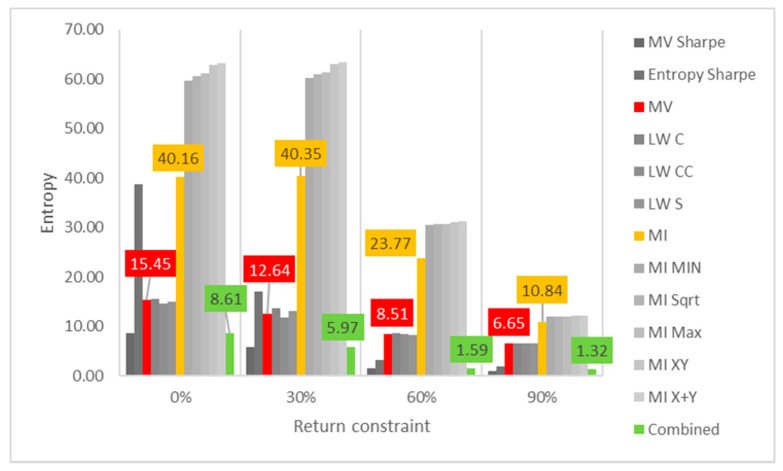
Entropy x return constraint for daily Bovespa return series; series appear in the same sequence of the data labels. Colored series denote MV, ME and combined models.

**Figure 8:**
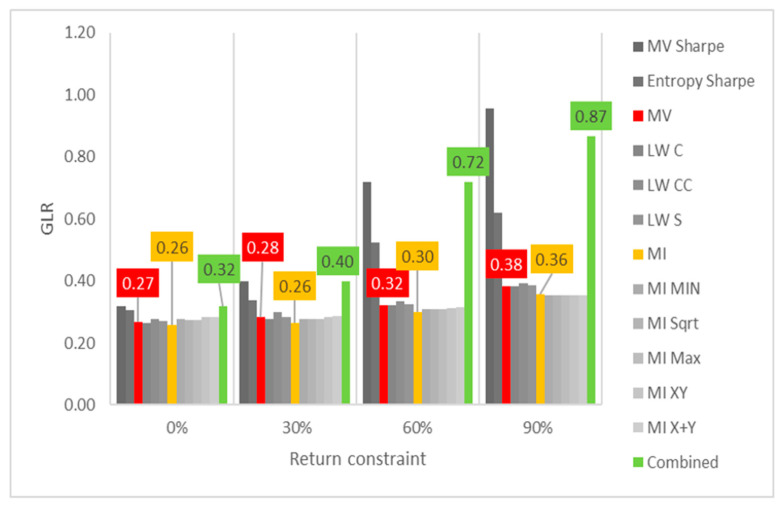
GLR x return constraint for daily Bovespa return series; series appear in the same sequence of the data labels. Colored series denote MV, ME and combined models.

When diversity measures are considered, the results are similar to those observed in the DJIA case. Entropy decreases sharply for ME models, while MV and robust models only see minor decreases, as observed in Figure 7 and Figure 8. In terms of GLR, both ME, MV and robust models tend to increase it for each increment in the return constraint. Sharpe variants of these models are more sensitive to increases in the return constraint.

- S&P500

Considering the daily returns of 395 S&P500 constituent stocks from January 2015 to December 2019, with 839 days being used to train the model and 419 for testing the output weights while imposing no return constraints (in that case, minimizing variance and entropy). The results are shown in Table 6.

MV and robust models show better results than ME, naïve and Sharpe variants of these models, while also remaining less volatile with lower observed standard deviation and thus culminating in higher Sharpe ratios. In terms of portfolio diversity though, ME models have an edge in comparison to MV and robust models, not only in terms of entropy (more invested securities) but also showing fewer correlation adjusted concentrations, while Sharpe models perform the worst. Here, the combined model allocates almost all its resources in the MV Sharpe model, as has been the case in most of the previous tests.

For the monthly return test, where 40 months are used to train the model and 20 to test while also imposing no return constraint, the following results in Table 7 were achieved:

Here, the situation seems to be reversed from what was the case in the daily results, i.e., ME models outperform MV and robust ones, while also showing fewer standard deviation and higher Sharpe ratios. In contrast, ME models still are more dispersedly distributed when compared to MV and robust ones, but now they show higher correlation adjusted concentration. Sharpe variants of these models perform poorly on every measure. The combined portfolio was able to find a solution allocating its resources on all available models, as was the case with monthly Bovespa returns.

To conclude this section, the model’s response to change in the return constraint was analyzed utilizing its daily returns series, while increasing the return constraints from 0% to 30% with 10% increment steps, as was done in the DJIA case.

**Figure 9:**
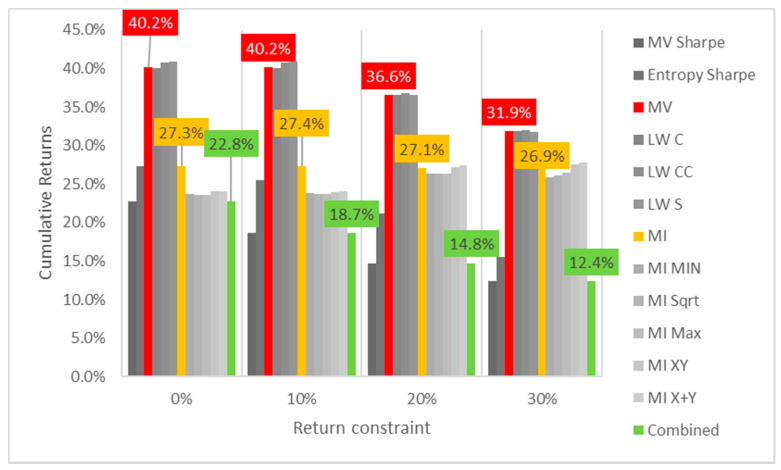
Cumulative returns x return constraint for daily S&P500 return series; series appear in the same sequence of the data labels. Colored series denote MV, ME and combined models.

**Figure 10:**
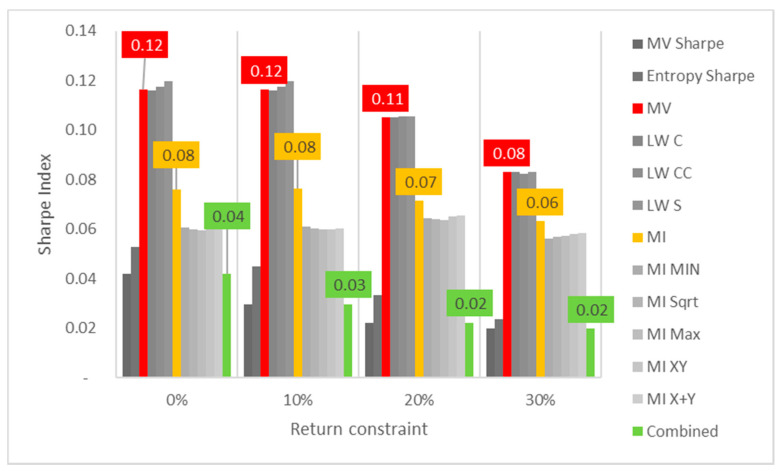
Sharpe index x return constraint for daily S&P500 return series; series appear in the same sequence of the data labels. Colored series denote MV, ME and combined models.

Figure 9 and Figure 10 report the cumulative return x return constraint as well as Sharpe index x return constraint for daily S&P500 return series. Here, the results are similar to what was observed with the DJIA case, i.e., an increase in the return constraint actually diminishes performance for out-of-sample tests, with the exception that ME models seem to increase their performance a little, but with the caveat of diminishing Sharpe ratios, indicating higher volatilities. Sharpe variations of the models are more sensitive to an increase in the return constraint, and thus show steeper decreases in performance when compared to their standard counterparts.

Next, the sensitivity of diversity measures was tested.

**Figure 11:**
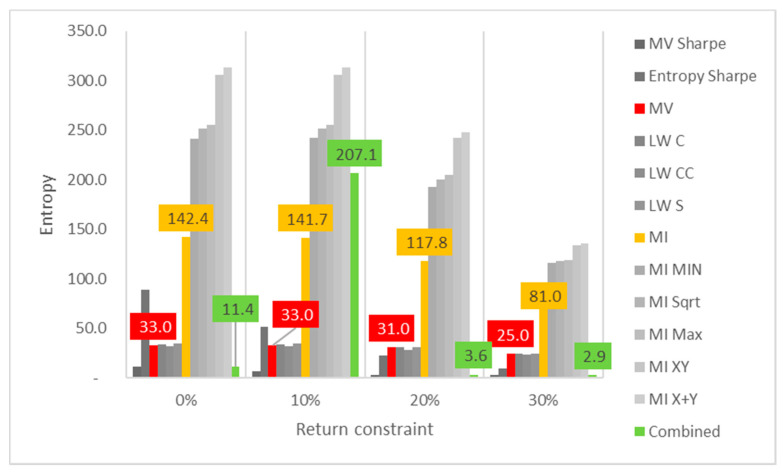
Entropy x return constraint for daily S&P500 return series; series appear in the same sequence of the data labels. Colored series denote MV, ME and combined models.

**Figure 12:**
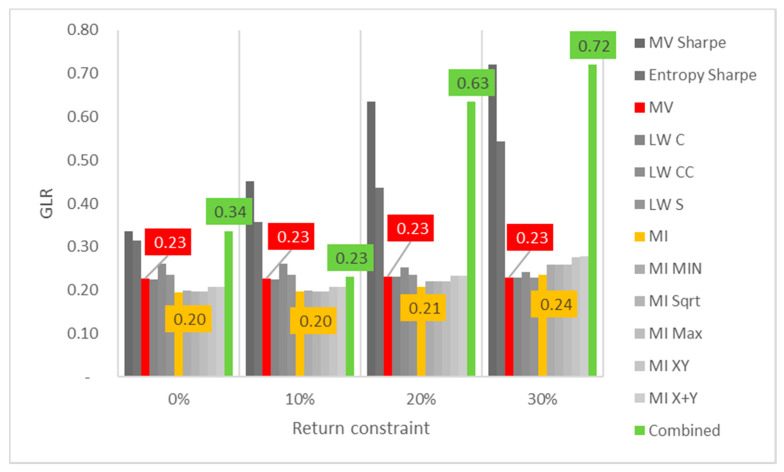
GLR x return constraint for daily S&P500 return series; series appear in the same sequence of the data labels. Colored series denote MV, ME and combined models.

Figure 11 and Figure 12 show the Entropy x return constraint and GLR x return constrant for daily S&P return series. In this case, it is also evident that the increase in the return constraint decreases portfolio diversification, as it can be observed by the steep decline in portfolio entropy and increase in portfolio correlation adjusted concentration, more apparent in ME and Sharpe variants of standard models. MV and robust models show lower sensitivity to this increase, as their resulting portfolios were already more concentrated in the first place.

- Comparing results between tests

Observing the results in the previous sections of this study, it is noticeable that there is little uniformity between them and that the model’s performance behaves differently for each dataset. To compare the models’ results between all tests and assess the overall performance of each model, the ranking of each model/variable combination was measured for each test, considering decreasing order for cumulative returns, Jensen’s alpha, p1, p99, Sharpe and entropy and increasing order for standard deviation and GLR. In this case, the best performing model was ranked first and the worst 14th for each variable. The results were then averaged between the different tests and the results from daily return series are show in Table 8 below (lowest is best).

The standard ME model with the unnormalized MI was the best ranking model when considering the average of all variables for daily results. Its main strength lies in its GLR measure, which was consistently the lowest in all datasets tested, while showing average performance for other variables. The Ledoit Wolf single market factor model ranked second overall, being the top performer in cumulative returns, Jensen’s measure, standard deviation, P1 and Sharpe, though showing poor results for entropy, GLR and P99 measures. In general, MV and robust models tend to perform better in terms of cumulative returns, standard deviations and P1, hence also showing better Sharpe ratios overall. ME models instead show better performance in diversity measures, such as portfolio entropy and GLR, with P99 (abnormal returns) being another positive highlight for these models.

Sharpe variations of ME and MV models show the best performances in P99 measure but fall short on all others. MV Sharpe specially is the worst performer in all but three variables, while ME Sharpe is still raked top 3 in terms of cumulative returns and Jensen’s measure but is the worst performer in terms of standard deviation and P1 (abnormal losses). It is also important to notice that all standard ME and MV models are better ranked than naïve diversified portfolios, while the combined model showed the second worst rank overall due to its tendency of concentrating its weights in the Sharpe variants of ME and MV models.

For the monthly return series, the results are shown in Table 9 below. 

First, it is important to highlight the main difference between these results with the previous one shown in Table 8, i.e., ME models now perform better overall than MV and robust, except when considering GLR and P1 measures. The best ranking models are concentrated on normalized variants of MI, while the standard MI was ranked second worst. Robust models perform better than MV on all measures but P99, and Ledoit Wolf constant shrinkage method stands out as the best performing robust method, with the best average GLR ranking.

Naïvely diversified portfolios were also better ranked than MV and Sharpe models, indicating a reduction in efficiency when utilizing monthly return series in comparison to daily returns. Combined portfolios now show better results, with the differential evolution solver being able to spread more evenly its weights between the different models, instead of concentrating its weights in one solution as in the previous case. Please see Table A1 in the Appendix A for detail.

## 5. Conclusions

The present work aimed to propose and compare a new portfolio optimization model using entropy as a measure of risk, considering 5 years of data of DJIA, Bovespa and S&P500 indexes’ constituents comparing a range of performance and diversity measures for the resulting portfolios utilizing daily and monthly return series. It was found that ME models do not always outperform their MV and robust counterparts, although presenting an edge in terms of portfolio diversity measures, especially for portfolio weight entropy. For daily returns, ME models ranked similarly with robust LW methods across a range of performance measures, and standard MI was able to consistently deliver better diversified portfolios than all other methods in terms of correlated adjusted concentration. Considering monthly returns, ME models ranked better than MV and robust methods overall, and a higher tendency to concentrate portfolios was observed on all models, even in terms of GLR. When increasing return constraints on portfolio optimization, ME models were more stable overall, showing dampened responses in cumulative returns and Sharpe indexes in comparison to MV and robust methods, but concentrated their portfolios more rapidly as they were more evenly spread initially. It was also shown that, depending on the market, increasing return constraints may have a positive or negative impact on the out-of-sample performance, as it was observed on the Bovespa and DJI cases, respectively. This finding suggests that earlier contradictions in the literature regarding the benefits of estimating and usage of the return vector, where Ledoit [5,7,11] find that the minimum variance portfolio performs best out-of-sample while others such as Yu et al. 2014 suggest otherwise, might be explained by the dataset being utilized. It is also important to highlight that DeMiguel et al., 2009 affirm that MV models dominate naïve portfolios when high levels of idiosyncratic volatility are observed, but the results in this work suggest otherwise: MV models had far better results in the S&P and DJIA markets than naïve portfolios, but worse on the Bovespa case, a historically more volatile market than the aforementioned ones.

Although the present research aimed to validate the proposed models considering different conditions of initial constraints, varying datasets, time intervals, and separating train and test observations, it is limited in other dimensions. First, as was shown in this work, some conclusions can be different depending on the dataset tested, so general conclusions cannot be drawn immediately and the application of the model needs to be addressed case by case. Second, the combined portfolio method only maximized return, very often concentrating weights in the best performing in sample model, which not always translated into the best performer out-of-sample. Third, weight rebalancing was not implemented, and a substantial part of the literature analyzes performance of automated portfolios with varying sets of rebalancing windows and transactional costs. Finally, other performance measures and statistical tests might be considered to jointly evaluate the performance of a set of portfolio optimizing models.

As ME models proposed in this work focus on changing the risk variable being utilized without fundamentally changing MV’s structure, future research could focus on implementing current improvements proposed to MV models for ME. In that case, the addition of real-life constraints, dynamic programming for continuously rebalancing portfolios and the utilization of fuzzy logic to estimate future returns might bring even better results to ME models. Other opportunities are present on changing the objective function of the combined portfolio optimizer to consider other relevant variables, enhancing its out-of-sample performance. Finally, the discretization process of the return series can also be improved by implementing more advanced density estimation techniques.

## Figures and Tables

**Table 1:** Label and corresponding model.

Label	Model
MV Sharpe	Mean-Variance Sharpe
Entropy Sharpe	Mean-Entropy Sharpe
MV	Mean-Variance
LW C	Ledoit Wolf Constant
LW CC	Ledoit Wolf Constant Correlation
LW S	Ledoit Wolf Single Market Factor
MI	Standard Mean-Entropy
MI MIN	MI normilized by the minimum entropy
MI Sqrt	MI normilized by the square root of multiplied entropies
MI Max	MI normilized by the maximum entropy
MI XY	MI normilized by the joint entropy
MI X+Y	MI normilized by the sum of X and Y entropies
Naive	Naive portfolio
Combined	Combined portfolio optimization

**Table 2:** DJIA daily returns test; Alfa refers to Jensen’s measure.

	Performance						Diversity	
Models	Return	Alfa	Stdev	P1	P99	Sharpe	Entropy	GLR
LW S	44.3%	0.4%	0.0072	−2.0%	1.7%	0.114	8.86	0.41
MV	43.8%	-	0.0072	−2.0%	1.6%	0.113	9.05	0.41
LW C	43.5%	−0.3%	0.0071	−1.9%	1.6%	0.113	9.52	0.40
LW CC	41.8%	−1.8%	0.0071	−1.9%	1.6%	0.109	9.87	0.40
MI	35.7%	−4.4%	0.0078	−2.4%	2.0%	0.086	25.69	0.33
MI MIN	34.1%	−5.6%	0.0082	−2.6%	2.0%	0.079	28.26	0.35
MI Sqrt	34.1%	−5.6%	0.0082	−2.6%	2.0%	0.079	28.27	0.35
MI Max	34.1%	−5.7%	0.0082	−2.6%	2.0%	0.079	28.27	0.35
MI XY	33.6%	−6.1%	0.0083	−2.6%	2.0%	0.077	28.65	0.36
MI X+Y	33.6%	−6.2%	0.0083	−2.6%	2.0%	0.077	28.68	0.36
Entropy Sharpe	32.9%	−5.5%	0.0100	−2.9%	2.6%	0.065	18.70	0.43
Naive	32.5%	−6.9%	0.0086	−2.7%	2.1%	0.072	29.00	0.37
Combined	26.0%	−11.0%	0.0099	−2.6%	2.5%	0.052	4.49	0.44
MV Sharpe	26.0%	−11.0%	0.0099	−2.6%	2.5%	0.052	4.48	0.44

**Table 3:** DJIA monthly returns test; Alfa refers to Jensen’s measure.

	Performance						Diversity	
Models	Return	Alfa	Stdev	P1	P99	Sharpe	Entropy	GLR
MV	48.4%		0.0298	−6.2%	5.9%	0.624	8.30	0.32
LW C	45.3%	−5.1%	0.0320	−6.3%	6.3%	0.583	12.11	0.30
LW S	44.7%	−3.9%	0.0291	−6.0%	5.8%	0.595	7.90	0.35
MV Sharpe	37.8%	−17.3%	0.0422	−8.1%	7.9%	0.362	4.29	0.45
Combined	37.8%	−17.3%	0.0421	−8.1%	7.9%	0.362	4.31	0.45
LW CC	37.4%	−12.4%	0.0320	−6.9%	6.4%	0.460	6.99	0.44
Entropy Sharpe	31.8%	−19.8%	0.0410	−7.4%	7.8%	0.316	11.77	0.36
Naive	31.1%	−23.8%	0.0376	−7.5%	7.1%	0.334	29.00	0.34
MI X+Y	30.5%	−24.0%	0.0367	−7.4%	6.9%	0.334	28.38	0.34
MI XY	29.9%	−24.0%	0.0360	−7.4%	6.7%	0.334	26.98	0.34
MI MIN	29.9%	−23.8%	0.0358	−7.3%	6.7%	0.336	26.89	0.34
MI MAX	29.9%	−24.3%	0.0363	−7.4%	6.8%	0.332	27.04	0.34
MI Sqrt	29.9%	−24.1%	0.0361	−7.4%	6.7%	0.333	27.04	0.34
MI	24.2%	−30.3%	0.0379	−7.6%	6.2%	0.259	7.06	0.43

**Table 4:** Bovespa daily returns test; Alfa refers to Jensen’s measure.

	Performance						Diversity	
Models	Return	Alfa	Stdev	P1	P99	Sharpe	Entropy	GLR
Entropy Sharpe	79.9%	43.1%	0.0140	−3.4%	3.3%	0.087	38.85	0.31
Naive	66.0%	30.0%	0.0129	−3.1%	2.9%	0.078	65.00	0.29
MI X+Y	61.7%	26.4%	0.0123	−3.0%	2.7%	0.076	63.14	0.28
MI XY	61.6%	26.3%	0.0123	−3.0%	2.7%	0.076	62.91	0.28
MI MAX	60.2%	25.2%	0.0120	−2.9%	2.7%	0.076	61.13	0.27
MI Sqrt	59.8%	24.8%	0.0120	−2.9%	2.7%	0.076	60.57	0.27
MI MIN	59.2%	24.3%	0.0119	−2.9%	2.7%	0.075	59.64	0.28
Combined	53.1%	18.4%	0.0124	−2.6%	2.8%	0.065	8.61	0.32
MV Sharpe	53.1%	18.4%	0.0124	−2.6%	2.8%	0.065	8.60	0.32
MI	49.6%	16.1%	0.0108	−2.6%	2.5%	0.068	40.16	0.26
LW C	32.4%	0.1%	0.0097	−2.2%	2.1%	0.044	15.68	0.27
MV	32.2%	-	0.0097	−2.2%	2.1%	0.044	15.45	0.27
LW S	32.1%	−0.1%	0.0097	−2.2%	2.1%	0.043	15.00	0.27
LW CC	32.0%	−0.4%	0.0097	−2.2%	2.1%	0.043	14.70	0.28

**Table 5:** Bovespa monthly returns test; Alfa refers to Jensen’s measure.

	Performance						Diversity	
Models	Return	Alfa	Stdev	P1	P99	Sharpe	Entropy	GLR
Entropy Sharpe	108.5%	91.6%	0.0661	−7.8%	20.2%	0.501	13.42	0.28
Naive	63.5%	45.3%	0.0607	−10.0%	14.2%	0.336	65.00	0.30
MI X+Y	58.8%	40.7%	0.0592	−9.6%	14.2%	0.318	59.97	0.33
MI MAX	57.3%	39.2%	0.0586	−9.5%	14.2%	0.312	52.59	0.35
MI Sqrt	54.9%	36.8%	0.0582	−9.3%	14.1%	0.301	46.69	0.37
MI MIN	54.6%	36.5%	0.0585	−9.4%	14.2%	0.297	43.46	0.38
MI XY	54.5%	36.4%	0.0583	−9.3%	14.2%	0.298	42.87	0.38
Combined	41.9%	23.2%	0.0570	−9.2%	13.5%	0.228	34.26	0.32
MI	41.6%	23.3%	0.0608	−9.3%	15.0%	0.214	5.63	0.55
LW C	40.0%	21.5%	0.0647	−7.4%	13.0%	0.224	19.36	0.29
LW S	33.4%	14.5%	0.0575	−7.9%	13.9%	0.171	13.32	0.36
LW CC	30.6%	11.0%	0.0647	−9.1%	15.7%	0.142	6.88	0.50
MV Sharpe	21.5%	2.4%	0.0590	−9.6%	13.6%	0.088	5.92	0.41
MV	19.6%	-	0.0665	−11.1%	14.6%	0.073	6.74	0.44

**Table 6:** S&P500 daily returns test; Alfa refers to Jensen’s measure.

	Performance						Diversity	
Models	Return	Alfa	Stdev	P1	P99	Sharpe	Entropy	GLR
LW S	40.9%	1.2%	0.0063	−1.7%	1.4%	0.120	35.43	0.24
LW CC	40.7%	0.6%	0.0064	−1.8%	1.4%	0.118	32.30	0.26
MV	40.2%	-	0.0064	−1.8%	1.4%	0.116	33.02	0.23
LW C	40.1%	−0.2%	0.0064	−1.8%	1.3%	0.116	33.79	0.23
Entropy Sharpe	27.4%	−16.5%	0.0103	−3.3%	2.7%	0.053	88.99	0.32
MI	27.3%	−11.7%	0.0068	−2.2%	1.6%	0.076	142.45	0.20
MI X+Y	24.1%	−15.7%	0.0076	−2.5%	1.8%	0.060	313.62	0.21
MI XY	24.0%	−15.8%	0.0076	−2.5%	1.8%	0.060	306.02	0.21
MI MIN	23.7%	−15.7%	0.0074	−2.4%	1.7%	0.061	241.30	0.20
Naive	23.7%	−17.2%	0.0085	−2.7%	1.9%	0.054	395.00	0.26
MI Sqrt	23.6%	−15.9%	0.0075	−2.4%	1.7%	0.060	251.49	0.20
MI MAX	23.5%	−16.1%	0.0076	−2.4%	1.8%	0.059	255.18	0.20
Combined	22.8%	−20.9%	0.0112	−3.1%	3.3%	0.042	11.44	0.34
MV Sharpe	22.8%	−20.9%	0.0112	−3.1%	3.3%	0.042	11.42	0.34

**Table 7:** S&P500 daily returns test; Alfa refers to Jensen’s measure.

	Performance						Diversity	
Models	Return	Alfa	Stdev	P1	P99	Sharpe	Entropy	GLR
MI MIN	31.3%	16.4%	0.0339	−7.2%	6.8%	0.368	80.91	0.35
MI XY	31.1%	16.2%	0.0339	−7.1%	6.8%	0.366	92.90	0.35
MI Sqrt	31.1%	16.2%	0.0339	−7.1%	6.8%	0.366	90.79	0.35
MI X+Y	30.9%	15.5%	0.0350	−7.3%	7.3%	0.353	166.12	0.33
MI MAX	30.7%	15.7%	0.0341	−7.2%	6.9%	0.360	110.11	0.34
MI	28.0%	13.2%	0.0341	−7.1%	7.1%	0.329	12.19	0.41
Combined	27.5%	10.8%	0.0374	−8.0%	7.4%	0.298	146.70	0.30
LW CC	27.3%	11.6%	0.0362	−8.2%	6.2%	0.304	22.36	0.32
LW S	26.6%	9.1%	0.0391	−8.8%	7.4%	0.278	46.16	0.25
LW C	23.4%	4.0%	0.0362	−9.1%	8.5%	0.224	51.18	0.26
Naive	22.4%	3.0%	0.0467	−9.2%	9.4%	0.202	395.00	0.34
Entropy Sharpe	20.4%	−3.5%	0.0719	−14.6%	12.4%	0.140	17.14	0.50
MV	20.1%	-	0.0466	−9.2%	9.3%	0.181	17.96	0.27
MV Sharpe	14.1%	−7.5%	0.0526	−11.3%	8.2%	0.118	14.24	0.36

**Table 8:** Daily return series tests’ model rankings.

Models	Return	Alfa	Stdev	P1	P99	Sharpe	Entropy	GLR	AVG
MV Sharpe	12.3	11.7	13.0	9.7	2.3	12.7	13.0	14.0	11.1
MV	5.7	-	2.3	2.7	12.7	5.7	9.7	7.0	6.5
LW C	6.0	5.3	2.3	3.0	13.7	6.0	8.7	6.0	6.4
LW CC	6.7	6.0	2.3	2.3	11.3	6.7	10.0	9.0	6.8
LW S	5.0	4.7	2.0	2.0	12.3	5.0	9.7	8.0	6.1
Entropy Sharpe	5.7	5.3	13.3	14.0	1.7	8.3	7.0	12.0	8.4
MI	7.0	6.0	5.0	5.0	10.0	6.0	6.0	1.0	5.8
MI MIN	7.3	6.0	6.7	7.3	7.7	6.3	5.0	5.0	6.4
MI Sqrt	8.0	7.0	7.0	7.7	8.0	7.3	4.0	4.0	6.6
MI Max	8.3	7.3	7.3	8.0	8.3	7.7	3.0	3.0	6.6
MI XY	7.0	6.7	9.3	10.3	5.7	6.7	2.0	6.3	6.8
MI X+Y	6.7	6.3	9.7	11.3	5.3	7.0	1.0	7.3	6.8
Naive	8.0	8.0	11.7	12.3	3.3	8.0	-	9.3	8.7
Combined	11.3	10.7	12.0	9.3	2.7	11.7	12.0	13.0	10.3

**Table 9:** Monthly return series tests’ rankings.

Models	Return	Alfa	Stdev	P1	P99	Sharpe	Entropy	GLR	AVG
MV Sharpe	10.3	10.0	11.3	12.7	6.0	11.0	12.3	12.3	10.8
MV	9.3	-	9.0	9.0	6.7	9.0	9.7	5.7	8.3
LW C	7.3	7.3	7.0	4.7	9.7	7.3	6.7	1.7	6.5
LW CC	8.7	7.3	7.0	5.3	8.7	7.7	10.0	10.0	8.1
LW S	7.7	7.0	4.3	4.3	10.7	7.3	8.7	5.7	7.0
Entropy Sharpe	6.7	6.3	13.0	8.3	1.7	9.0	8.7	8.3	7.8
MI	9.7	9.0	8.7	6.7	8.0	10.0	12.0	12.7	9.6
MI MIN	6.0	4.7	4.0	6.0	10.0	5.0	5.0	8.0	6.1
MI Sqrt	7.0	6.3	3.7	5.7	9.7	6.3	3.7	8.3	6.3
MI Max	7.0	6.7	6.0	7.7	7.0	6.7	2.3	7.3	6.3
MI XY	6.3	6.3	4.3	5.0	8.3	5.7	4.3	8.3	6.1
MI X+Y	5.3	5.7	7.7	9.3	7.3	5.3	1.0	4.7	5.8
Naive	7.0	7.0	10.3	12.0	4.3	7.7	-	5.0	7.6
Combined	6.7	7.3	7.7	8.3	7.0	7.0	6.7	7.0	7.2

## Data Availability

The data to support the findings of the current study are available upon request from the corresponding author.

## Appendix A

**Table A1:** Combined portfolio weights by test runs; on all daily test runs, the optimization routine was unable to distribute weights between all available portfolios, while in monthly runs for Bovespa and S&P all portfolios were invested.

		MI SQRT	MI MAX	MV	MI XY	MI X+Y	MI MIN	Naive	MI	MV Sharpe	Entropy Sharpe	LW S	LW C	LW CC
Daily	DJIA	0.00%	0.00%	0.00%	0.00%	0.00%	0.00%	0.00%	0.00%	99.95%	0.02%	0.00%	0.00%	0.00%
	Bovespa	0.00%	0.00%	0.00%	0.00%	0.00%	0.00%	0.00%	0.00%	99.97%	0.02%	0.00%	0.00%	0.00%
	S&P	0.00%	0.00%	0.00%	0.00%	0.00%	0.00%	0.00%	0.00%	99.98%	0.01%	0.00%	0.00%	0.00%
Monthly	DJIA	0.00%	0.00%	0.00%	0.00%	0.00%	0.00%	0.00%	0.00%	99.93%	0.04%	0.00%	0.00%	0.00%
	Bovespa	6.27%	1.63%	12.17%	2.19%	1.42%	1.18%	27.56%	0.02%	13.52%	0.13%	20.56%	5.49%	7.87%
	S&P	20.75%	1.11%	0.64%	12.70%	6.38%	11.07%	8.33%	6.55%	5.54%	0.28%	15.71%	7.38%	3.57%
